# Mix and match. A simulation study on the impact of mixed-treatment comparison methods on health-economic outcomes

**DOI:** 10.1371/journal.pone.0171292

**Published:** 2017-02-02

**Authors:** Pepijn Vemer, Maiwenn J. Al, Mark Oppe, Maureen P. M. H. Rutten-van Mölken

**Affiliations:** Institute for Medical Technology Assessment (iMTA), Erasmus University, Rotterdam, The Netherlands; Public Library of Science, FRANCE

## Abstract

**Background:**

Decision-analytic cost-effectiveness (CE) models combine many parameters, often obtained after meta-analysis.

**Aim:**

We compared different methods of mixed-treatment comparison (MTC) to combine transition and event probabilities derived from several trials, especially with respect to health-economic (HE) outcomes like (quality adjusted) life years and costs.

**Methods:**

Trials were drawn from a simulated reference population, comparing two of four fictitious interventions. The goal was to estimate the CE between two of these. The amount of heterogeneity between trials was varied in scenarios. Parameter estimates were combined using direct comparison, MTC methods proposed by Song and Puhan, and Bayesian generalized linear fixed effects (GLMFE) and random effects models (GLMRE). Parameters were entered into a Markov model. Parameters and HE outcomes were compared with the reference population using coverage, statistical power, bias and mean absolute deviation (MAD) as performance indicators. Each analytical step was repeated 1,000 times.

**Results:**

The direct comparison was outperformed by the MTC methods on all indicators, Song’s method yielded low bias and MAD, but uncertainty was overestimated. Puhan’s method had low bias and MAD and did not overestimate uncertainty. GLMFE generally had the lowest bias and MAD, regardless of the amount of heterogeneity, but uncertainty was overestimated. GLMRE showed large bias and MAD and overestimated uncertainty. Song’s and Puhan’s methods lead to the least amount of uncertainty, reflected in the shape of the CE acceptability curve. GLMFE showed slightly more uncertainty.

**Conclusions:**

Combining direct and indirect evidence is superior to using only direct evidence. Puhan’s method and GLMFE are preferred.

## 1. Introduction

In 2006, The Netherlands implemented conditional reimbursement of potentially innovative, but expensive hospital drugs, on the condition that further real-life evidence is collected.[1] After four years, a new reimbursement decision is made, based on all evidence available. Unfortunately, new drugs are often compared to placebo or standard care and the interventions of interest vary by country or over time. Trials incorporating all competing interventions are impractical at best, impossible at worst.[2] This means that a direct, head-to-head comparison may not be available. If a comparison via a common comparator is available, an indirect treatment comparison (ITC) can be used to combine the relative effects of the two treatments versus the a common comparator.[3] With three or more interventions, there may be direct evidence for some pairs of interventions, while other pairs can be compared only via one or more of the other interventions. Techniques to analyze all the available evidence simultaneously are called mixed treatment comparisons (MTC).

To aid reimbursement decision making, a probabilistic decision-analytic cost-effectiveness (CE) model is often used, using parameters that are calculated from evidence combined using meta-analysis. The choice of meta-analysis method can considerably affect final CE estimates.[4] Most studies comparing meta-analysis methods focused on a single treatment effect (e.g. [5–8]) or made a qualitative comparison (e.g. [9]). However, in modeling studies a wide range of model parameters need to be estimated.[10] In this study we aimed the following:

*To compare the performance of standard methods of MTC when applied to different types of model parameters, especially with respect to their impact on health-economic (HE) outcomes*.

We answered this question, by performing a simulation study. This paper is structured as follows. First, the setup of the simulation study is discussed in section 2.1, followed by the disease and model structure used, in 2.2. In 2.3, we then discus the scenarios that have been used to analyze different amounts of heterogeneity between trials. The methods of meta-analysis compared in this study are discussed in 2.4, followed by the indicators to compare the performance of these methods in 2.5. The results are divided in outcomes on individual model input parameters (3.1 and 3.2) and HE-outcomes (3.3). Section 4 contains a discussion of the results as well as a conclusion. A similar comparison of direct meta-analysis methods is reported separately.[11]

## 2. Methods

### 2.1. Simulation study

**Fig 1:**
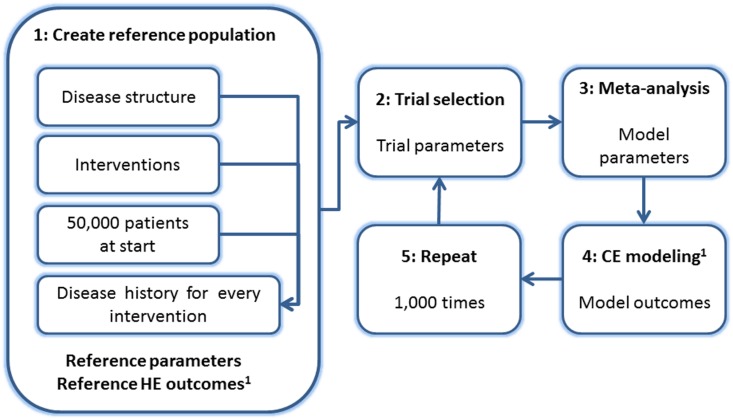
Design of the simulation study. HE = Health-economic, CE = Cost-effectiveness.

In order to compare standard methods of MTC we have performed a simulation study. The set-up of the simulation study is presented in this section. The simulation study, as well as the disease and HE model structure as discussed in section 2.2, is identical to that used in the earlier publication.[11] The simulation consists of five steps (Fig 1). In **step 1: Create reference population**, we simulated a superpopulation [12] containing 50,000 patients. The disease progression was simulated four times for each patient, once for each of four fictitious interventions. The mean values of parameters and HE outcomes within this reference population represent the ‘truth’ with which parameter estimates and HE outcomes were compared, referred to as *reference parameters* and *reference outcomes*. Parameters included transition and event probabilities, maintenance and event costs, utilities and utility-decrements due to an event. HE outcomes included (quality adjusted) life years (QALY/LY), intervention and maintenance costs, number of events, incremental CE ratio (ICER) and CE acceptability curves (CEAC).

In **step 2: Trial selection**, we sampled trials comparing two treatments from the reference population. For each of the trials we calculated *trial parameters*. In **step 3: Meta-analysis** we calculated *model parameters*, by pooling trial parameters using several methods of meta-analysis (section 3.4). We used a CE model in **step 4: CE modeling**, which was filled with a set of model parameters obtained by each of the methods of meta-analysis. Based on the transition probabilities obtained in step 3, patients change from one disease stage to the next. Costs and utilities, also estimated in step 3, are counted for each cycle in a disease stage. These can be summed over life time, which provides an estimate of the total costs and health outcomes for each of the interventions that one wishes to compare. Probabilistic sensitivity analysis (PSA; 1,100 iterations), which takes into account parameter uncertainty, yielded *model outcomes*.

To study systematic differences between the methods of meta-analysis, we repeated steps 2 to 4 in **step 5: Repeat** in 1,000 *repetitions*.

A complete overview of the software used in this study, as well as the outcomes of the simulation study, can be found online at https://figshare.com/projects/Mix_and_match_-_A_simulation_study_on_the_impact_of_mixed-treatment_comparison_methods_on_health-economic_outcomes/13438.

### 2.2. Disease and model structure

**Fig 2:**
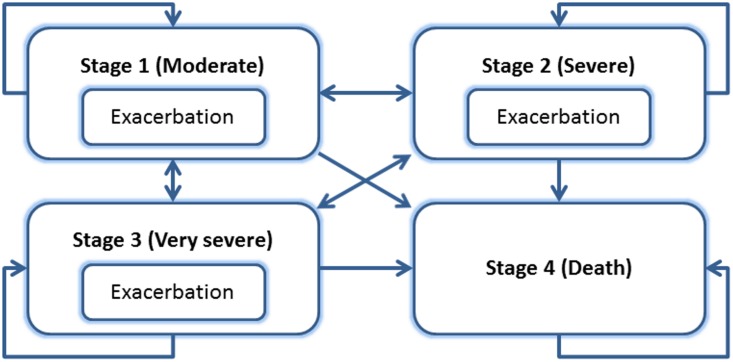
Design of the chronic disease model.

In this section we discuss the specification of the disease and the structure of the HE model. The structure is representative for common health economic models for chronic diseases that is characterized by progression and the occurrence of temporary events, during which symptoms temporarily worsen, simulated using a four-stage Markov model (Fig 2). The structure is identical to the structure used in an earlier publication.[11]

To simulate disease progression, we first defined the Reference Disease Progression (RDP), which can be thought of as the disease progression of an untreated, base-case patient. The RDP was modified based on individual patient characteristics and interventions, to simulate a heterogeneous population of individual patients. Table 1 shows the characteristics of the reference population that was simulated. By sampling from sub-populations, it was possible to add heterogeneity to trials in relevant scenarios.

**Table 1:** Characteristics of the simulated patient population.

Total number of patients	50,000
Starting disease stage	5/8 in Moderate, 2/8 in Severe and 1/8 in Very Severe
Gender	50% male, 50% female
Age in years	18–34; 35–64; 65+
	Determined by a random draw from a uniform distribution from 18 to 75
Developed/developing country.	50% from developed countries, 50% developing countries
Body Mass Index (BMI)	<25 (average or low); 25–30 (high); >30 (obese),
	Determined by a random draw from a normal distribution with mean 23 and standard deviation of 4.
Smoking status	30% smokers, 70% non-smokers

The way patient characteristics and interventions influenced the RDP is stated in S1 Table.

Focusing on the interventions, “No Intervention” has no effects on the RDP. “Old Intervention” decreases the probability of an event and has a positive effect on mortality, with a one-off cost of €250 at the beginning of treatment. “Usual Care” decreases the probability of disease progression at €60 per month. “New Intervention” costs €350 per month and decreases the probability of disease progression, increases the probability of moving to a better disease stage and decreases the probability of an event. The intervention effects are dependent on the disease stage of the patient.

Changes to parameters were additive across patient characteristics and interventions. For example, for a female patient aged 35–64 who gets New Intervention, the probability to move from the severe disease stage to death was 10% (RDP) -2% (modification for gender) +4% (age) -3% (intervention) = 9%.

The comparison of interest is that between New Intervention and Usual Care. Table 2 shows the reference outcomes when applying these interventions to the complete patient population.

**Table 2:** Reference outcomes for Usual Care and New Intervention, per patient after 12 cycles—Mean (Standard deviation)^a^.

Variables	Usual Care	New Intervention	Difference
QALYs^b^	0.485 (0.232)	0.540 (0.231)	0.054
LYs^b^	0.740 (0.328)	0.786 (0.313)	0.046
Intervention costs	€530 (€240)	€3,300 (€1,310)	€2770
Maintenance costs	€3,260 (€2,080)	€3,070 (€1,810)	- €180
Event costs	€2,330 (€2,610)	€1,260 (€1,780)	- €1070
Total costs	€6,120 (€4,340)	€7,630 (€3,830)	€1520
Number of cycles in:			
Moderate disease	5.171 (3.750)	6.209 (3.965)	1.038
Severe disease	2.477 (2.512)	2.313 (2.507)	-0.164
Very severe disease	1.238 (1.850)	0.911 (1.554)	-0.327
Death	3.114 (3.937)	2.567 (3.751)	-0.547
Number of events	1.160 (1.259)	0.630 (0.856)	-0.530
Proportion surviving	49.9%	58.3%	8.4%pt
ICER, total costs per QALY^b^			€28,020

^a^ ICER for other comparisons: New Intervention versus Old Intervention €30,440; Usual Care versus Old Intervention €42,760; New Intervention versus No Intervention €21,830; Usual Care versus No Intervention €13,750; Old Intervention versus No Intervention €3,680. Mean and standard deviation for costs were rounded to nearest €10.

^b^ LY = life year, QALY = quality adjusted LY, ICER = incremental Cost-effectiveness Ratio

The structure of the HE model mirrors the disease progression. We assumed that trial data was collected each month during one year. Likewise, the time horizon of the HE model was 1 year, with monthly cycles. We did not apply discounting. Simulation and modeling was performed using SAS 9.2 and WinBUGS 1.4.3.

### 2.3. Scenarios

**Fig 3:**
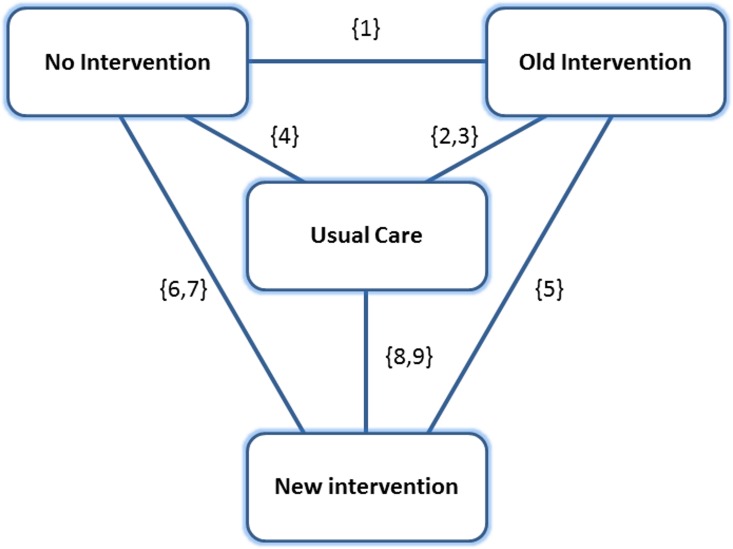
Evidence network for simulation study. The figures in curly brackets are the trial numbers making the corresponding comparisons, as described in the text. Trials 1, 6 and 8 are trials that may be drawn from a subpopulation in selected scenarios.

The amount of heterogeneity in the trials sampled in **step 2: Trial selection** was varied in eight scenarios. We discuss these scenarios in this section. Heterogeneity in the meta-analysis literature is any kind of variability in outcomes between different studies,[13] which is caused by both aleatoric uncertainty (the intrinsic uncertainty of a phenomenon) and (unknown or unmeasured) underlying differences between the study characteristics. We used the mechanism as discussed in section 2.2 to create this systematic heterogeneity between patients. All scenarios contained data from nine trials, with 500 patients in each of the two treatment arms. The comparisons made in each of the trials can be found in Fig 3. It is clear from this graph that the nine trials provide evidence for all available contrasts. A similar structure can be found in Hasselblad [14] and Lu and Ades.[15]

The heterogeneity in all eight scenarios is described in Table 3. In scenario 8 we used heterogeneity definitions at extreme values. This scenario is included as a stress test for the methods, with extreme amounts of heterogeneity between trials. In practice, trials that display this amount of heterogeneity would (should) not be combined.

**Table 3:** Overview of heterogeneity of different scenarios in the simulation study. Scenarios discussed in detail in the main text are in bold. Other scenarios are primarily shown in the appendix.

Scenario	Added heterogeneity with effect on disease progression
**1**	**9 randomly drawn trials, with 500 patients in each of the treatment arms.**
2	8 randomly drawn trials
	Non-random trial 1 (Old Intervention versus No Intervention), with worse average health.^a^
3	8 randomly drawn trials
	Non-random trial 6 (New Intervention versus No Intervention), with worse average health.^a^
**4**	**8 randomly drawn trials**
	**Non-random trial 8 (New Intervention versus Usual Care directly), with worse average health.**^a^
5	7 randomly drawn trials
	Non-random trial 1 (Old Intervention versus No Intervention), with worse average health.^a^
	Non-random trial 6 (New Intervention versus No Intervention), with lower average age.^b^
6	6 randomly drawn trials
	Non-random trial 1 (Old Intervention versus No Intervention), with worse average health.^a^
	Non-random trial 6 (New Intervention versus No Intervention), with lower average age.^b^
	Non-random trial 8 (New Intervention versus Usual Care directly), with higher average age.^c^
**7**	**6 randomly drawn trials**
	**Non-random trials 1 (Old Intervention versus No Intervention), 6 (New Intervention versus No Intervention) and 8 (New Intervention versus Usual Care), with worse average health.**^a^
8	6 randomly drawn trials
	Non-random trials 1 (Old Intervention versus No Intervention), 6 (New Intervention versus No Intervention) and 8 (New Intervention versus Usual Care), with worse average health.^a^
	Extreme scenario

^a^ Trial contains, on average, patients with a higher age, more smokers and more obesity; patients have therefore on average a more rapid disease deterioration, higher event probability, higher maintenance costs, lower quality of life.

^b^ Trial contains, on average, patients with a lower age; patients have therefore on average a slower disease deterioration.

^c^ Trial contains, on average, patients with a higher age; patients have therefore on average a more rapid disease deterioration.

### 2.4. Methods of meta-analysis

In this section, we discuss the methods of meta-analysis to be included in this study. We first discuss combining only the available direct evidence in the network. This is a relevant comparison, since it has been debated whether or not direct and indirect evidence can and should be combined, or even if indirect methods should be used at all. Since there is no reason not to use direct evidence when it is available, results on indirect treatment comparison methods, disregarding direct evidence, were not reported separately in this paper. The four methods of indirect meta-analysis compared in this study are the methods proposed by Song et al. [16], the logistic regression approach proposed by Puhan et al. [17] and Strassman et al. [18], and the Bayesian generalized linear model, either in a fixed effect or random effect specification.[19,20]

All methods attempt to calculate ${\hat{\delta }}_{jk}$, estimates of the relative difference between two treatments *j* and *k* = 1,…, *K*, *δ*_*jk*_. Despite being used in many applications of MTC, the odds ratio (OR) is not commonly used in HE modeling. We have chosen to use the natural logarithm of the relative risk ln(*RR*) as relative measure of treatment benefit for the transition and event probabilities. For all non-relative variables in the model -costs, quality of life weights and baseline values for the comparator-, we used estimates obtained with the DerSimonian-Laird random effects method (DL).[21]

This method is also used as a baseline method, to combine all available direct evidence (DIRECT) on the difference between New Intervention and Usual Care in the network. The pooled estimate ${\hat{\delta }}_{jk}^{DL}$, in this case on a ln(*RR*) scale, is calculated as a weighted average of individual study estimates, using the inverse of the within-study and between-study variance (heterogeneity) as weights. We denote an estimate of *δ*_*jk*_ from study *s* = 1,…, *S* by ${\hat{\delta }}_{s}$, and its precision, defined as the reciprocal of the estimate’s within-study variance, by *w*_*s*_. The model is assumed to be

$${\hat{\delta }}_{s}\sim N\left(\mu_{s}\left(\delta \right),v_{s}\right),\;\mu_{s}\left(\delta \right)\sim N\left(\mu \left(\delta \right),\tau_{{}_{i}}^{2}\right)$$ (1)

An estimate of the between-study heterogeneity $\tau_{{}_{i}}^{2}$ is obtained from:

$${\hat{\tau }}^{2}=\{\begin{array}{ll} \frac{\sum w_{s}{\left(\delta_{s}-{\hat{\delta }}_{jk}^{FE}\right)}^{2}-\left(S-1\right)}{\sum w_{s}-\frac{\sum w_{s}^{2}}{\sum w_{s}}} & ,\sum w_{s}{\left(\delta_{s}-{\hat{\delta }}_{jk}^{FE}\right)}^{2}\geq \left(S-1\right)\\ 0 & ,\sum w_{s}{\left(\delta_{s}-{\hat{\delta }}_{jk}^{FE}\right)}^{2}<\left(S-1\right) \end{array}$$ (2)

where ${\hat{\delta }}_{jk}^{FE}$ is the fixed effect estimate of *δ*_*jk*_, calculated as a weighted average of the individual study estimates, using only *w*_*s*_ as weights. The DL estimate incorporates ${\hat{\tau }}_{{}_{i}}^{2}$ into the weights:

$${\hat{\delta }}_{jk}^{DL}=\frac{\sum w_{s}^{*}{\hat{\delta }}_{s}}{\sum w_{s}^{*}},\;w_{s}^{*}={\left(w_{s}^{-1}+{\hat{\tau }}^{2}\right)}^{-1}$$ (3)

The variance of the DL-estimate ${\hat{v}}_{jk}^{DL}$ is calculated as.

$${\hat{v}}_{jk}^{DL}=\frac{1}{\sum w_{i}^{*}}$$ (4)

The first MTC method in our study was proposed by Song et al. (SONG).[16] If direct evidence is available between baseline intervention 1 and intervention of interest *k*, a direct estimate ${\hat{\delta }}_{1k}^{DL}$ is calculated for the difference between the two interventions, using the DL method described above. Next, all possible indirect estimates, via intermediate interventions denoted as *j*, are calculated.[22] For any combination for which direct evidence is available between interventions 1 and *j*, and *k* and *j*, the estimate of indirect association on a ln(*RR*) scale ${\hat{\delta }}_{1k}^{j}$ is calculated as

$${\hat{\delta }}_{1k}^{j}={\hat{\delta }}_{1j}^{DL}-{\hat{\delta }}_{kj}^{DL}$$ (5)

The paired comparisons of 1 versus *j*, ${\hat{\delta }}_{1j}^{DL}$, and *k* versus *j*, ${\hat{\delta }}_{kj}^{DL}$ are calculated using the DL-method, The estimated variance of ${\hat{\delta }}_{1k}^{j}$ can be obtained from

$${\hat{v}}_{1k}^{j}={\hat{v}}_{1j}^{DL}+{\hat{v}}_{kj}^{DL}$$ (6)

The SONG estimate ${\hat{\delta }}_{1k}^{SONG}$ of the association between 1 and *k*, in our study Usual Care and New Intervention, is then calculated by performing a DL meta-analysis, where each direct and indirect estimate and its estimated variance, is treated as if it were a single trial.[16]

The second method in our study was proposed by Puhan et al., who performed a fixed effect logistic regression (PUHAN) [17,18]:

$$\text{log}\left(\frac{p_{i}}{1-p_{i}}\right)=\beta +\sum_{k=2}^{K}\delta_{1k}Tr_{ki}+\sum_{s=2}^{S}\lambda_{s}St_{si}$$ (7)

A data set is first created, based on summary tables from each included study. The number of data entries is equal to the number of patients in each respective study, with treatment variables *Tr*_*k*_,*k* = 2,…,*K* as independent dummy variables. To preserve randomization within each trial, a study dummy variable *St*_*s*_,*s* = 2,…,*S* was also included. This dummy variable also adjusts for differences in patient profiles and study setup between trials.[17] Intervention 1 and study 1 are treated as baseline. The binomial dependent variable was whether or not patient *i* made the transition, or experienced the event that was being modeled. The parameter estimate ${\hat{\delta }}_{1k}$ belonging to treatment *k* can be interpreted as the ln(*OR*) of the event occurring between interventions 1 and *k*. We estimated ${\hat{\delta }}_{1k}^{PUHAN}$ on a ln(*RR*) scale and its estimated variance, using the ln(*OR*) estimates from the model, and the treatment effects of the baseline intervention. See S1 Text for the calculation used.

The generalized linear model (GLM) is the most widely used method of MTC, and is also applicable for direct meta-analysis.[19,20] Where frequentist methods such as DIRECT, SONG and PUHAN implicitly assume a normal distribution, Bayesian GLM allows the definition of many different possible link functions, depending on the nature of the data. The fixed effects specification of the GLM (GLMFE) requires the trial data, the definition of a prior for the parameter of interest and a likelihood function linking both. Defining *n*_*sk*_ as the number of events, out of the total number of patients in each arm *n*_*sk*_, for intervention arm *k* of study *s*, we assumed that the data generation process follows a binomial likelihood:

$$r_{sk}\sim Binomial\left(p_{sk},n_{sk}\right)$$ (8)

where *p*_*sk*_ represents the probability of an event in arm *k* of trial *s*. We modeled the probabilities of events *p*_*sk*_ on the logit-scale, the most commonly used link function for a binomial likelihood [19]:

$$\text{log}\left(\frac{p_{sk}}{1-p_{sk}}\right)=\mu_{s}+\delta_{s,1k}*I\left(k\neq 1\right)$$ (9)

where *I*(*k* ≠ 1) takes the value 0 when intervention *k* is equal to baseline intervention 1, and 1 otherwise, *μ*_*s*_ is the trial-specific log-odds in the comparator arm, and *δ*_*s*,1*k*_ is the trial-specific log OR of events for treatment group *j* compared to baseline intervention 1. Notice the similarities between the GLM specification, and PUHAN.

For the random effects specification (GLMRE), we assumed

$$\delta_{s,1k}\sim N\left(\delta_{1k},\sigma^{2}\right)$$ (10)

where *σ*^2^ represents the between-trial heterogeneity. Note that the heterogeneity variance is assumed to be the same between different treatment comparisons; a necessary restriction unless there are large numbers of studies per comparison. For the GLMFE, (Eq 8) reduces to

$$\text{log}it\left(p_{sk}\right)=\mu_{s}+\delta_{1k}*I\left(k\neq 1\right)$$ (11)

which is equivalent to setting *σ*^2^ in (Eq 10) to zero, thus assuming homogeneity of the underlying treatment effects. The GLM procedure calculates a posterior estimate for ${\hat{\delta }}_{1k}$ on a log OR scale. Using a different link function and therefore directly calculating outcomes on a ln(*RR*) scale is possible, but may run into computational problems.[23] We therefore estimated ${\hat{\delta }}_{1k}^{GLM}$ on a ln(*RR*) scale and its dispersion parameter, using the ln(*OR*) estimates from the model, and the treatment effect of the baseline intervention.

We used a flat beta prior *Beta*(0.5,0.5) for all baseline transitions, and a flat normal prior *N*(0,1*E*12) for all other baseline parameters. We used a flat normal prior centered on *N*(0,1*E*8) for all treatment effects of the comparator. For GLMRE, we used the inverse of a squared uniform distribution *U*(0.001,10) for 1/*σ*^2^ where *σ*^2^ is the between-trial heterogeneity from (Eq 10). The minimum value of this prior was not 0, to avoid numerical problems.

Conceptually, confidence intervals in frequentist statistics and credibility intervals in Bayesian statistics have very different interpretations (e.g. [24,25]). However, for convenience and legibility, we abbreviate both as CI. For each pooled parameter estimate, we report the mean and the 95% CI. Interested readers may request code on both the simulation study and the methods of meta-analysis from the corresponding author.

### 2.5. Comparing performance

In this section, we discuss the metrics to compare the statistical performance of the methods. We assumed that a researcher doing a meta-analysis aims to estimate the CE of the New Intervention compared to Usual Care in the entire patient population, not a specific subgroup. Evidence on other interventions is solely used to provide extra evidence for this comparison. We further assumed that the researcher is unaware of the fact that heterogeneity, when present, was caused by sampling from subgroups. To the researcher, heterogeneity is either caused by random sampling or unobserved trial differences. These assumptions are made, because if these differences in design are known, either the trials would not be synthesized at all, or a way has to be found to control for these differences. These assumptions made it possible to judge the performance of the different methods of meta-analyses by comparing model parameters and HE outcomes with the reference values. Because the same patients were included to calculate HE outcomes for each method of meta-analysis, any difference between the methods can be attributed to the methods themselves (moderately independent simulations).[12]

Statistical performance is measured using coverage, statistical power, bias and mean absolute deviation (MAD). Coverage is the percentage of all repetitions, that the simulated CI covered the ‘truth’. Since the coverage is based on 95% CIs, we would expect that, if all trials are drawn randomly, the coverage should on average be close to 95%.[5,12,26] Over-coverage, where the CI are so wide that coverage rates are above 95 per cent, suggests the results are too conservative, which leads to a loss of statistical power. Under-coverage, coverage rates lower than 95 per cent, indicates over-confidence in the estimates, which leads to higher than expected type I error, since more simulations will incorrectly detect a significant result.[12] We said a method underestimated uncertainty if the coverage was smaller than 90%; and overestimated if the coverage was higher than 98%.

Statistical power is the percentage of all repetitions where the simulated result yields a statistically significant difference between the two treatments. Bias is the difference between the point estimate in the simulated data set and the true population value, averaged over all repetitions. MAD is the average, over all repetitions, of the absolute value of the bias. The MAD indicates how far the estimated value was from the ‘truth’, regardless of whether it was too high or too low.

## 3. Results

### 3.1. Parameter estimates for one set of trials

**Fig 4:**
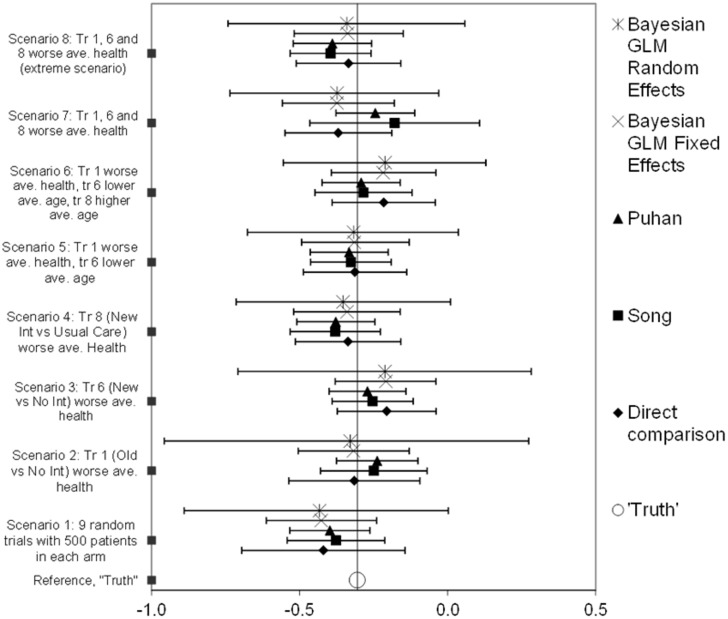
Meta-analysis on the logarithm of the risk ratio of the transition from the severe to very severe disease stage, for the New Intervention arm compared to the Usual Care arm, for one repetition. All scenarios have nine trials, each with 500 patients in both treatment arms.

We first compare the methods of meta-analysis on one example parameter for each of the scenarios, using only the first repetition (Fig 4). From bottom to top, we compare the different meta-analysis models for the eight scenarios. Each dot represents the point estimate for the parameter, in this case the transition probability from severe to very severe disease, and the bars the estimated CIs. The ‘true’ population value is displayed at the bottom. As can be seen, when all trials were drawn randomly (scenario 1), GLMRE had the broadest CI, followed by DIRECT, GLMFE and SONG. PUHAN had the smallest CI. All methods had the true parameter value in its CI and the point estimates were all very similar. In the other scenarios, each with a different amount of heterogeneity, we see a similar pattern as in scenario 1, except that in scenario 7 SONG had a relatively larger CI. The point estimate of SONG and PUHAN, and of GLMFE and GLMRE are very similar.

Based on similar patterns for other parameters (not shown), we can conclude that DIRECT and GLMRE yielded the widest CI. GLMFE had a point estimate that is generally closer to the true parameter value than DIRECT, with a smaller CI. The smallest CI was found for SONG and PUHAN. In all scenarios, for all methods, the true parameter value lay within the CI of the estimated parameters.

### 3.2. Parameter estimates for 1,000 repetitions

The results from the previous section might be due to chance. To see if there were systematic differences, we now discuss parameter estimates averaged over 1,000 repetitions. Table 4 shows the number of parameters that correspond to several threshold values of coverage, bias and MAD for three scenarios. Information on other scenarios can be found in S2 Table. No parameter had a mean coverage below 90%, which we defined as underestimation of uncertainty. The least overestimation of uncertainty could be found with DIRECT and PUHAN, regardless of the amount of heterogeneity.

**Table 4:** Summary of the results of meta-analysis on parameters of the health-economic model, which require network meta-analysis for three of the eight scenarios. Means over 1,000 repetitions.

	Scenario 1	Scenario 4	Scenario 7
Total number of parameters	12	12	12
Parameters influenced by added heterogeneity	0	9	9
Heterogeneity in the following trials	-	Trial 8 (New Int vs Usual)	Trial 1 + Trial 6 + Trial 8
Total number of parameters for which:			
Mean coverage < 90% (underestimation of uncertainty)			
Direct comparison (DIRECT)	0	0	0
Song’s method (SONG)	0	0	0
Puhan’s method (PUHAN)	0	0	0
Bayesian GLM FE method (GLMFE)	0	0	0
Bayesian GLM RE method (GLMRE)	0	0	0
Mean coverage > 98% (overestimation of uncertainty)			
DIRECT	1	0	2
SONG	11	4	7
PUHAN	3	1	3
GLMFE	10	6	4
GLMRE	12	12	12
Mean bias 1%-2%			
DIRECT	2	0	0
SONG	0	1	1
PUHAN	0	2	1
GLMFE	4	0	1
GLMRE	1	1	1
Mean bias > 2%			
DIRECT	0	9	9
SONG	0	8	8
PUHAN	0	8	8
GLMFE	1	9	9
GLMRE	9	10	11
Mean MAD^a^ 4%-7%			
DIRECT	5	4	4
SONG	8	8	8
PUHAN	8	9	9
GLMFE	5	4	4
GLMRE	4	4	4
Mean MAD^a^ > 7%			
DIRECT	6	7	7
SONG	1	2	3
PUHAN	0	0	0
GLMFE	7	8	8
GLMRE	8	8	8

^a^ MAD = Mean absolute deviation, minimum found is 2.6%.

In scenario 1, GLMRE had a large number of parameters with an average bias larger than 1% or even 2%. All methods had a large number of parameters with a large bias in scenario 4, where extra heterogeneity was added to trial 8, which directly compares the Usual Care with the New Intervention. In scenario 7, where three out of nine trials have patients drawn from a subpopulation, all methods showed bias in several parameters. The lowest amount of bias was found in SONG and PUHAN, with a similar number of parameters in each category of bias.

For all methods, the estimated parameter value was quite far from the true population value. The minimum MAD, averaged over 1,000 estimates of the same parameters (not in graphs/tables), ranged from 2.6% for PUHAN to 4.2% for GLMRE. In other words, none of the methods estimated parameters with an average MAD lower than 2.6%. The maximum MAD, averaged over 1,000 estimates of the parameters, was 14.4% for GLMRE in scenario 7. This means that one of the parameters, in this case ln(*RR*) of the number of events in the severe disease stage, differed from the reference value by more than 14%, averaged over 1,000 repetitions. The discrepancy will therefore be much larger for individual repetitions. SONG and PUHAN generally had the lowest number of parameters in each of the categories of MAD.

Generally, SONG, GLMFE and GLMRE overestimated uncertainty for most parameters. PUHAN overestimated uncertainty for fewer parameters. Neither of these methods underestimated uncertainty. The bias and MAD was generally lowest for SONG and PUHAN, followed by GLMFE.

### 3.3. Health-economic outcomes for 1,000 repetitions

After having compared the methods of meta-analysis on the parameters, we now turn our attention to the HE outcomes. In Table 5, we show the coverage, statistical power, bias and MAD for three scenarios. Information on other scenarios can be found in S3 Table. It shows the range in values over the four types of HE outcomes, the difference in QALYs, LYs, number of events and total costs. PUHAN had a coverage closest to the benchmark of 95%. Only in case of heterogeneity (scenario 7) did PUHAN overestimate uncertainty. Both GLMFE and GLMRE had a coverage above 99% for all methods, for all HE outcomes. No method underestimated uncertainty.

**Table 5:** Coverage, statistical power, absolute value of the bias and mean absolute deviation (MAD) of health-economic outcomes for three of the eight scenarios.

	Direct comparison	Song’s method	Puhan’s method	GLM FE method	GLM RE method
Coverage, range in values over the four health-economic outcomes^a^					
Scenario 1: Nine randomly drawn trials	>98%	>98%	97.0%-97.3%	>98%	100%
Scenario 4: Eight randomly drawn trials; one trial drawn from a less health population	97.1%-98.6%	>98%	96.8%-97.9%	>99%	100%
Scenario 7: Six randomly drawn trials; three trials drawn from a less healthy population	97.2%-99.1%	97.9%-99.3%	96.3%-98.2%	>99%	100%
Statistical power, range in values over the four health-economic outcomes^a^					
Scenario 1: Nine randomly drawn trials	81.5%-100%	95.3%-100%	>99%	73.4%-100%	5.8%-95.9%
Scenario 4: Eight randomly drawn trials; one trial drawn from a less healthy population	76.3%-100%	93.5%-100%	>98%	56.8%-100%	4.1%-94.3%
Scenario 7: Six randomly drawn trials; three trials drawn from a less healthy population	79.3%-100%	91.9%-100%	>98%	60.3%-100%	3.6%-93.5%
Bias, range in values over the four health-economic outcomes^a^					
Scenario 1: Nine randomly drawn trials	0.4%-5.7%	0.2%-3.0%	0.2%-2.1%	0.3%-3.5%	0.3%-13.6%
Scenario 4: Eight randomly drawn trials; one trial drawn from a less healthy population	0.5%-11.8%	0.5%-5.5%	0.5%-5.4%	0.8%-9.3%	2.0%-6.3%
Scenario 7: Six randomly drawn trials; three trials drawn from a less healthy population	0.2%-10.1%	0.5%-9.7%	0.4%-8.1%	0.0%-7.7%	0.2%-17.8%
MAD, range in values over the four health-economic outcomes^a^					
Scenario 1: Nine randomly drawn trials	6.0%-21.7%	5.1%-17.9%	4.9%-16.9%	6.2%-22.7%	6.9%-25.9%
Scenario 4: Eight randomly drawn trials; one trial drawn from a less healthy population	6.6%-23.5%	5.3%-18.4%	5.1%-17.4%	6.8%-25.1%	7.9%-29.9%
Scenario 7: Six randomly drawn trials; three trials drawn from a less healthy population	6.3%-22.8%	5.4%-19.2%	5.1%-18.0%	6.8%-24.1%	7.9%-28.5%

^a^ QALYs, LYs, number of events and total costs

Regardless of heterogeneity, GLMRE had the lowest statistical power. For the difference in LYs, GLMRE had a statistical power below 10% in scenario 1, where all trials were drawn randomly, and even lower in scenarios with added heterogeneity. All methods had a statistical power of 100% for the number of events and above 99% for total costs, in all scenarios. PUHAN generally had the lowest bias and MAD across all scenarios. GLMRE had the highest MAD for all HE outcomes in all scenarios. In S3 Table, the results for the different HE outcomes are presented separately.

**Fig 5:**
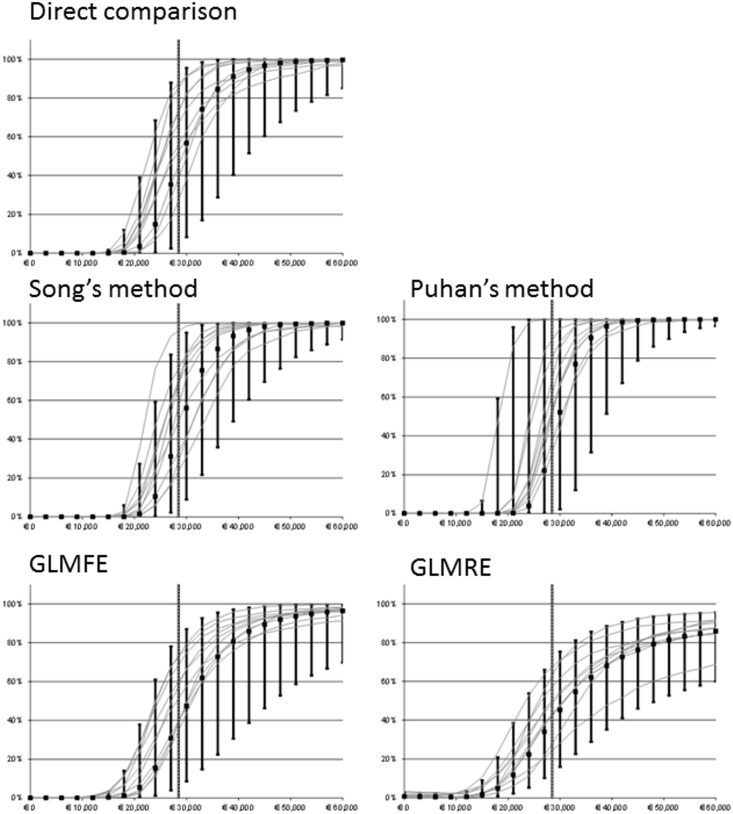
Cost-effectiveness acceptability curves (CEACs) for the five meta-analysis methods in the heterogeneous scenario 7. The vertical lines depicts median, 2.5th and 97.5th percentile of the likelihood that the New Intervention is cost-effective compared with Usual care, at various threshold values of a QALY (averaged over 1,000 repetitions). The curves are the CEACs for the first 10 repetitions. The dotted vertical line is the ‘true’ population ICER.

In Fig 5 we show the CE acceptability curves (CEACs) for scenario 7 where three trials are drawn from a less healthy population. The five graphs represent the methods we compared. In each graph, we show the CEAC of ten repetitions, the median and 2.5^th^ and 97.5^th^ percentiles over 1,000 repetitions. The vertical line indicates the true population ICER. Graphs for other scenarios can be found in S1 Fig. Ideally, the methods would show a low value for the CEAC below the true population ICER, and a high value above.

In this scenario, we can see that SONG and PUHAN displayed a steeper shape than the other methods. This indicates that they were more certain of the CE of the New Intervention than the other methods. At a WTP of €30,000 per QALY, which is close, but slightly above the true population ICER, the median likelihood that the New Intervention was cost-effective was 56% for SONG, 52% for PUHAN, 47% for GLMFE and 45% for GLMRE. At higher WTPs, GLMFE and GLMRE were less certain than the other methods. At a WTP of €60,000 per QALY, the median probability of the New Intervention being accepted was 100% for SONG and PUHAN, 96% for GLMFE and 86% for GLMRE. At a WTP of €60,000 per QALY, the difference between the 2.5^th^ percentile and the 97.5^th^ percentile was 9 percentage points (%pt, 100%-91%) for SONG. For PUHAN this difference was 3%pt, for GLMFE 29%pt and for GLMRE 36%pt. With less heterogeneity (scenario 1 and 4), the CEACs express a higher certainty for all methods. Still, SONG and PUHAN seem to be the most certain of the CE, in these scenarios followed by GLMFE. SONG and PUHAN still had the most certainty around the CE. SONG had all the CEACs lying closest to each other.

Regardless of the amount of heterogeneity, SONG and PUHAN lead to the least amount of uncertainty. GLMFE model is slightly less certain. DIRECT and GLMRE have a lot of uncertainty, even at WTP values far from the true population ICER. They also display a lot of differences between the different repetitions.

## 4. Discussion

In this study, we compared four methods of indirect meta-analysis in a simulation study and judged their statistical performance by creating a gold standard. On a parameter level, Puhan’s method (PUHAN) showed the best performance, overestimating uncertainty for the fewest parameters with low bias and MAD. Song’s method (SONG) and the Bayesian fixed effect generalized linear model (GLMFE) also had generally low bias and MAD.

On HE outcomes, PUHAN showed a coverage closest to 95%, regardless of heterogeneity. Only with high heterogeneity did PUHAN overestimate uncertainty. Both PUHAN and GLMFE performed best on bias and MAD, followed by SONG. GLMFE had a very high coverage, which we defined as overestimating uncertainty. The same is true for the Bayesian random effect generalized linear model (GLMRE), which also had the lowest statistical power and the highest MAD for all HE outcomes.

The use of these methods would lead to differences in policy decisions. Using either only the direct evidence or GLMRE would lead to more rejections of new treatments compared to the other methods or more unnecessary research. Generally speaking, sophisticated methods require more data than simple methods, because of the increased number of parameters. It is possible that the GLMRE method, which requires the largest number of parameter to be estimated, may have more desirable properties when more trials have to be combined. Unfortunately, this situation is unlikely within the scope of the expensive drug program in the Netherlands. Based on this study, we would recommend either PUHAN or GLMFE. PUHAN is easier to implement and more easily understood by physicians and policy makers who will be using the results. GLMFE is the most widely used method, but requires advanced knowledge of statistical programming.

In scenarios, we covered many likely situations. We have drawn all trials randomly, added heterogeneity on the different “legs” of the network, and changed the amount of heterogeneity. One of the scenarios was “extreme” scenario 8, with trials that display such an amount of heterogeneity that in practice would not be combined. Although this lowered the practical applicability, it does give insight into the performance of the different methods (see Supporting Information for results). For example, no parameter had a mean coverage below 90% (section 3.2), except in scenario 8. In this scenario GLMFE and GLMRE had the least amount of parameters for which uncertainty was underestimated. The maximum MAD over all methods in this extreme scenario ranged from 17.6% for SONG, to 27.6% for GLMRE. Compared to a few large trials, the effect of having more but smaller trials and trials with differences in trial sizes, on the performance of different methods of meta-analysis is small.[11] We therefore feel our study results are generalizable to many other situations where parameters for a HE model are obtained through MTC.

However, the network is very “regular” with direct evidence for all treatment combinations. This is often not the case. New interventions are usually only compared to the latest alternative, or to placebo. Other forms of the evidence network are routinely found in MTC research. It remains open to further research how adding irregularity to such networks will change the results of this study. In particular, due to the regularity of the network, SONG could be used to full effect in our study, using information from all possible indirect comparisons. Since SONG can only be used in triangular networks, it is possible that not all available evidence can be used when applying SONG. This would for example be true in our study if no evidence existed on one of the treatment combinations. PUHAN, GLMFE and GLMRE would still be able to use all available data. This will likely diminish the performance of SONG compared with the other three methods.

Another limitation is the choice of prior for the Bayesian models. In the case of meta-analysis, a small number of studies is extra vulnerable to the type of prior.[8,27] As we did not assume the researcher to have prior information, we used vague priors. Even though they are supposed to be “uninformative”, they may influence posterior outcomes, especially for scale parameters.[27] We tested several different prior specifications but did not find any differences in outcomes.

Bayesian statistics at its heart is ideally suited for meta-analysis, since the premise of both are the same: prior available information is updated with new data.[28] However, the numerical method used for the Bayesian methods is not ideally suited for a simulation study such as we have done. Checking for convergence requires visual examination of plots, and careful examination of other outcome measures, which is infeasible when performing 1,000 repetitions. We therefore assumed that the MCMC procedure used to fit the Bayesian models achieved convergence in all simulations.

A final limitation is that no formal testing was performed on the consistency of the networks included.[29,30] Inconsistency can be thought of as a conflict between the direct and indirect evidence that is combined. Inconsistency, as with heterogeneity, is caused by an imbalance in the distribution of effect modifiers in the different arms.[29]

Within the MTC approach, evidence can be treated as a coherent whole, more data may be included, and in some cases the assumptions made in pair-wise approaches can be relaxed.[31] However, in practice, there is still a strong preference to use direct over indirect evidence. One of the main concerns is that indirect comparisons may be subject to greater biases than direct comparisons.[18] They are essentially observational findings across trials, and may have similar biases. The Cochrane Handbook for Systematic Reviews of Interventions recommends that direct and indirect evidence is considered separately and direct comparisons should take precedence as a basis for forming conclusions.[8] In contrast, it has also been argued that it would be improper to exclude any evidence.[32] Our study seems to support this second view: the direct comparison has a smaller statistical power, leading to new interventions not being found statistically different from older interventions. The biases and MAD are also higher than the MTC methods, except for the GLMRE method.

Heterogeneity in our reference population is modelled in a systematic manner, in order to see whether the methods would detect the heterogeneity that was introduced. If the heterogeneity would be drawn from a random distribution, as was done in for example [33], there is a possibility that a draw in one iteration would cancel the random draw in another iteration. By having heterogeneity in the same direction for each iteration, we could see how each method deals with this.

A crucial assumption is that the researcher performing the meta-analysis is not aware of these differences, as is very often the case in real life when heterogeneity is caused by unobserved factors.

If the confounding factor is unknown (e.g. genetics), the outcomes of heterogeneity tests might indicate “heterogeneity present” and the analysis might still be done. The latter is the case we are simulating, as we were interested in seeing whether the choice of method would impact reimbursement decisions.

In conclusion, when indirect evidence is available to inform a comparison between two interventions, regardless of the amount of heterogeneity present, combining all evidence is superior to using only the evidence from a head-to-head comparison of these two interventions. Puhan’s method and GLMFE showed similar results, with GLMFE having the tendency to overestimate uncertainty, but also having lower average bias and MAD. Based on this study, where we had to combine nine trials in a network that includes evidence for all treatment combinations, we would recommend PUHAN or GLMFE as the preferred method of indirect meta-analysis.

## Supporting information

S1 Fig Cost-effectiveness acceptability curves (CEACs). (DOCX)

S1 Table Reference Disease Progression. (DOCX)

S2 Table Results of meta-analysis on parameters. (DOCX)

S3 Table Health-economic outcomes. (DOCX)

S1 Text Converting Odds Ratio to Relative Risk. (DOCX)
