# Supplementary material for: Mix and match. A simulation study on the impact of mixed-treatment comparison methods on health-economic outcomes
Source: PLoS One. 2017 Feb 2;12(2):e0171292. doi: 10.1371/journal.pone.0171292 (PMC5289594; doi:10.1371/journal.pone.0171292)
Supplement: S1 Fig — (DOCX) [file pone.0171292.s001.docx]

S1 Fig Cost-effectiveness acceptability curves (CEACs)

Figure A1: Cost-effectiveness acceptability curves (CEACs) for the five meta-analysis methods in the heterogeneous scenario 1. Graphs depicts median, 2.5^th^ and 97.5^th^ percentile CEACs over 1,000 repetitions, as well as the CEACs for the first 10 repetitions; vertical line is the ‘true’ population ICER.

| Direct comparison   |  |
| --- | --- |
| Song   | Puhan   |
| GLM FE   | GLM RE   |

Figure A2: Cost-effectiveness acceptability curves (CEACs) for the five meta-analysis methods in the heterogeneous scenario 4. Graphs depicts median, 2.5^th^ and 97.5^th^ percentile CEACs over 1,000 repetitions, as well as the CEACs for the first 10 repetitions; vertical line is the ‘true’ population ICER.

| Direct comparison   |  |
| --- | --- |
| Song   | Puhan   |
| GLM FE   | GLM RE   |

Figure A3: Cost-effectiveness acceptability curves (CEACs) for the five meta-analysis methods in the heterogeneous scenario 8. Graphs depicts median, 2.5^th^ and 97.5^th^ percentile CEACs over 1,000 repetitions, as well as the CEACs for the first 10 repetitions; vertical line is the ‘true’ population ICER.

| Direct comparison   |  |
| --- | --- |
| Song   | Puhan   |
| GLM FE   | GLM RE   |
