# Supplementary material for: Mix and match. A simulation study on the impact of mixed-treatment comparison methods on health-economic outcomes
Source: PLoS One. 2017 Feb 2;12(2):e0171292. doi: 10.1371/journal.pone.0171292 (PMC5289594; doi:10.1371/journal.pone.0171292)
Supplement: S1 Table — (DOCX) [file pone.0171292.s002.docx]

S1 Table Reference Disease Progression

Table A1: Monthly transition probabilities in the Reference disease progression and changes in transition probabilities due to patient characteristics or interventions.

| From:/To: | Moderate | Severe | Very severe | Death |
| --- | --- | --- | --- | --- |
| Reference disease progression (RDP) | | | | |
| 1 – Moderate | 80% | 10% | 6% | 4% |
| 2 – Severe | 20% | 65% | 10% | 5% |
| 3 – Very Severe | 10% | 20% | 60% | 10% |
| 4 – Death | 0% | 0% | 0% | 100% |
| Changes due to gender. RDP = male, changes applicable to female patients. | | | | |
| 1 – Moderate | +1% | +1% | -1% | -1% |
| 2 – Severe | +1% | +1% | -1% | -1% |
| 3 – Very Severe |  | +1% | +1% | -2% |
| Changes due to age class. RDP = 18-34, changes applicable to patients aged 35-64, double these changes applicable to patients aged 65+. | | | | |
| 1 – Moderate | -4% | +2% |  | +2% |
| 2 – Severe |  | -4% | +2% | +2% |
| 3 – Very Severe |  |  | -4% | +4% |
| No changes due to developed/developing country. | | | | |
| Changes due to BMI. RDP = low/average BMI, changes applicable to patients with high BMI, double these changes applicable to patients with high BMI. | | | | |
| 1 – Moderate | -1% | +1% |  |  |
| 2 – Severe |  | -1% | +1% |  |
| 3 – Very Severe |  |  | -1% | +1% |
| Changes due to smoking status. RDP = non-smokers, changes applicable smokers. | | | | |
| 1 – Moderate | -3% | +1% | +1% | +1% |
| 2 – Severe |  | -3% | +1% | +2% |
| 3 – Very Severe |  |  | -3% | +3% |
| No changes due to use on No Intervention. | | | | |
| Changes due to use of Old Intervention | | | | |
| 1 – Moderate | +1% |  |  | -1% |
| 2 – Severe |  | +1% |  | -1% |
| 3 – Very Severe |  |  | +2% | -2% |
| Changes due to use of Usual Care. | | | | |
| 1 – Moderate | +5% | -2% | -2% | -1% |
| 2 – Severe |  | +2% | -1% | -1% |
| 3 – Very Severe |  |  | +2% | -2% |
| Changes due to use of New Intervention. | | | | |
| 1 – Moderate | +10% | -5% | -3% | -2% |
| 2 – Severe | +3% | +3% | -4% | -2% |
| 3 – Very Severe |  | +5% | -2% | -3% |

Table A2: Probability of an event per cycle for each disease stage in the Reference disease progression and changes in event probabilities due to patient characteristics or interventions.

| Disease stage | Moderate | Severe | Very severe | Death |
| --- | --- | --- | --- | --- |
| Reference disease progression (RDP) | 5% | 10% | 40% | 0% |
| No changes due to gender. |  |  |  |  |
| No changes due to age class. |  |  |  |  |
| No changes due to developed/developing country. |  |  |  |  |
| Changes due to BMI. RDP = low/average BMI, changes applicable to patients with high BMI, double these changes applicable to patients with double BMI. | +1% | +2% | +4% |  |
| Changes due to smoking status. RDP = non-smokers, changes applicable smokers. | +2% | +5% | +10% |  |
| No changes due to use of No Intervention. |  |  |  |  |
| Changes due to use of Old Intervention. |  |  |  |  |
| No changes due to use of Usual Care. | -1% | -2% | -10% |  |
| Changes due to use of New Intervention. | -2% | -10% | -20% |  |

Table A3: Monthly costs per stage using a Gamma distribution in the Reference disease progression and changes in distributional parameters due to patient characteristics or interventions.

| Disease stage | Moderate | Severe | Very severe | Death |
| --- | --- | --- | --- | --- |
| Reference disease progression (RDP) | | | | |
| Alpha | 4 | 5 | 10 |  |
| Beta | 50 | 80 | 100 |  |
| No changes due to gender. | | | | |
| Changes due to age class. RDP = 18-34, changes applicable to patients aged 35-64, double these changes applicable to patients aged 65+. | | | | |
| Alpha |  |  |  |  |
| Beta | +5 | +5 | +5 |  |
| Changes due to developed/developing country. RDP = developed country, changes applicable to patients from developing country. | | | | |
| Alpha |  |  |  |  |
| Beta | -10 | -10 | -10 |  |
| Changes due to BMI. RDP = low/average BMI, changes applicable to patients with high BMI, double these changes applicable to patients with double BMI. | | | | |
| Alpha |  |  |  |  |
| Beta | +2 | +2 | +2 |  |
| No changes due to smoking status. | | | | |
| No changes due to use of No Intervention. | | | | |
| No changes due to use of Old Intervention. | | | | |
| No changes due to use of Usual Care. | | | | |
| No changes due to use of New Intervention. | | | | |

Table A4: Quality of life weights using a Beta distribution in the Reference disease progression and changes in distributional parameters due to patient characteristics or interventions.

| Disease stage | Moderate | Severe | Very severe | Death |
| --- | --- | --- | --- | --- |
| Reference disease progression (RDP) | | | | |
| Alpha | 64 | 35 | 20 |  |
| Beta | 16 | 15 | 20 |  |
| No changes due to gender. | | | | |
| Changes due to age class. RDP = 18-34, changes applicable to patients aged 35-64, double these changes applicable to patients aged 65+. | | | | |
| Alpha | +5 | +5 | +5 |  |
| Beta | +5 | +5 | +5 |  |
| No changes due to developed/developing country. | | | | |
| Changes due to BMI. RDP = low/average BMI, changes applicable to patients with high BMI, double these changes applicable to patients with double BMI. | | | | |
| Alpha |  |  |  |  |
| Beta | +5 | +5 | +5 |  |
| No changes due to smoking status. | | | | |
| No changes due to use of No Intervention. | | | | |
| No changes due to use of Old Intervention. | | | | |
| No changes due to use of Usual Care. | | | | |
| No changes due to use of New Intervention. | | | | |

Table A5: Costs due to an event using a Gamma distribution in the Reference disease progression. No changes due to patient characteristics or interventions.

| Disease stage | Moderate | Severe | Very severe | Death |
| --- | --- | --- | --- | --- |
| Reference disease progression (RDP) | | | | |
| Alpha | 10 | 10 | 10 | 0 |
| Beta | 200 | 200 | 200 | 0 |

Table A6: Quality of life decrement due to an event using a Beta distribution in the Reference disease progression. No changes due to patient characteristics or interventions.

| Disease stage | 1 | 2 | 3 | 4 |
| --- | --- | --- | --- | --- |
| Reference disease progression (RDP) | | | | |
| Alpha | 6 | 6 | 6 | 0 |
| Beta | 4 | 4 | 4 | 0 |
