# Supplementary material for: Mix and match. A simulation study on the impact of mixed-treatment comparison methods on health-economic outcomes
Source: PLoS One. 2017 Feb 2;12(2):e0171292. doi: 10.1371/journal.pone.0171292 (PMC5289594; doi:10.1371/journal.pone.0171292)
Supplement: S2 Table — (DOCX) [file pone.0171292.s003.docx]

S2 Table Results of meta-analysis on parameters

Table A7: Summary of the results of meta-analysis on parameters of the health-economic model, which require network meta-analysis. Means over 1,000 repetitions.

|  | Scenario 1 | Scenario 2 | Scenario 3 | Scenario 4 | Scenario 5 | Scenario 6 | Scenario 7 | Scenario 8 |
| --- | --- | --- | --- | --- | --- | --- | --- | --- |
| Total number of parameters | 12 | 12 | 12 | 12 | 12 | 12 | 12 | 12 |
| Parameters influenced by added heterogeneity | 0 | 9 | 9 | 9 | 9 | 9 | 9 | 9 |
| Heterogeneity in the following trials | - | Trial 1 (Old Int vs No Int) | Trial 6 (New Int vs Old Int) | Trial 8 (New Int vs Usual) | Trial 1 + Trial 6 | Trial 1 + Trial 6 + Trial 8 | Trial 1 + Trial 6 + Trial 8 | Trial 1 + Trial 6 + Trial 8 |
| Total number of parameters for which: |  |  |  |  |  |  |  |  |
| Mean coverage < 90% (underestimation of uncertainty) |  |  |  |  |  |  |  |  |
| Direct comparison (DIRECT) | 0 | 0 | 0 | 0 | 0 | 0 | 0 | 4 |
| Song’s method (SONG) | 0 | 0 | 0 | 0 | 0 | 0 | 0 | 6 |
| Puhan’s method (PUHAN) | 0 | 0 | 0 | 0 | 0 | 0 | 0 | 7 |
| Bayesian GLM FE method (GLMFE) | 0 | 0 | 0 | 0 | 0 | 0 | 0 | 2 |
| Bayesian GLM RE method (GLMRE) | 0 | 0 | 0 | 0 | 0 | 0 | 0 | 0 |
| Mean coverage > 98% (overestimation of uncertainty) |  |  |  |  |  |  |  |  |
| DIRECT | 1 | 2 | 4 | 0 | 1 | 2 | 2 | 1 |
| SONG | 11 | 11 | 11 | 4 | 12 | 10 | 7 | 3 |
| PUHAN | 3 | 5 | 6 | 1 | 5 | 4 | 3 | 1 |
| GLMFE | 10 | 10 | 9 | 6 | 9 | 5 | 4 | 3 |
| GLMRE | 12 | 12 | 12 | 12 | 12 | 12 | 12 | 11 |
| Mean bias 1%-2% |  |  |  |  |  |  |  |  |
| DIRECT | 2 | 2 | 2 | 0 | 1 | 1 | 0 | 2 |
| SONG | 0 | 0 | 1 | 1 | 0 | 2 | 1 | 1 |
| PUHAN | 0 | 0 | 0 | 2 | 0 | 2 | 1 | 0 |
| GLMFE | 4 | 4 | 2 | 0 | 3 | 3 | 1 | 1 |
| GLMRE | 1 | 3 | 3 | 1 | 3 | 2 | 1 | 1 |
| Mean bias > 2% |  |  |  |  |  |  |  |  |
| DIRECT | 0 | 0 | 0 | 9 | 0 | 4 | 9 | 10 |
| SONG | 0 | 0 | 0 | 8 | 0 | 3 | 8 | 9 |
| PUHAN | 0 | 0 | 0 | 8 | 0 | 2 | 8 | 10 |
| GLMFE | 1 | 1 | 2 | 9 | 1 | 5 | 9 | 11 |
| GLMRE | 9 | 8 | 8 | 10 | 8 | 9 | 11 | 11 |
| Mean MAD^a^ 4%-7% |  |  |  |  |  |  |  |  |
| DIRECT | 5 | 7 | 5 | 4 | 6 | 4 | 4 | 3 |
| SONG | 8 | 9 | 9 | 8 | 9 | 7 | 8 | 3 |
| PUHAN | 8 | 8 | 8 | 9 | 8 | 8 | 9 | 3 |
| GLMFE | 5 | 5 | 5 | 4 | 5 | 5 | 4 | 2 |
| GLMRE | 4 | 4 | 4 | 4 | 4 | 4 | 4 | 2 |
| Mean MAD^a^ > 7% |  |  |  |  |  |  |  |  |
| DIRECT | 6 | 4 | 6 | 7 | 5 | 7 | 7 | 9 |
| SONG | 1 | 0 | 0 | 2 | 0 | 2 | 3 | 8 |
| PUHAN | 0 | 0 | 0 | 0 | 0 | 0 | 0 | 7 |
| GLMFE | 7 | 7 | 7 | 8 | 7 | 7 | 8 | 10 |
| GLMRE | 8 | 8 | 8 | 8 | 8 | 8 | 8 | 10 |

a MAD = Mean absolute deviation, minimum found is 2.6%.
