# Supplementary material for: Mix and match. A simulation study on the impact of mixed-treatment comparison methods on health-economic outcomes
Source: PLoS One. 2017 Feb 2;12(2):e0171292. doi: 10.1371/journal.pone.0171292 (PMC5289594; doi:10.1371/journal.pone.0171292)
Supplement: S3 Table — (DOCX) [file pone.0171292.s004.docx]

S3 Table Health-economic outcomes

Table A8 Health-economic outcomes for three of the eight scenarios. Means of coverage, bias and mean absolute deviance (MAD) of the difference between two interventions, over 1,000 repetitions.

| Scenario | Scenario 1 | | | | Scenario 2 | | | | Scenario 3 | | | |
| --- | --- | --- | --- | --- | --- | --- | --- | --- | --- | --- | --- | --- |
|  | Nine randomly drawn trials | | | | Eight randomly drawn trials; one trial drawn from a less healthy population | | | | Eight randomly drawn trials; one trial drawn from a less healthy population | | | |
|  | Coverage | Bias | MAD | Stat Power | Coverage | Bias | MAD | Stat Power | Coverage | Bias | MAD | Stat Power |
| Number of QALYs |  |  |  |  |  |  |  |  |  |  |  |  |
| Direct comparison | 98,9% | -3,6% | 13,0% | 99,6% | 97,5% | -4,2% | 13,1% | 99,2% | 97.9% | -3.7% | 13.1% | 99,6% |
| Song | 98,6% | -1,9% | 10,6% | 99,9% | 98,1% | -3,2% | 10,6% | 100,0% | 98.4% | -5.1% | 11.3% | 100.0% |
| Puhan | 97,2% | -1,3% | 10,0% | 100,0% | 96,7% | -1,9% | 10,0% | 100,0% | 96.4% | -4.1% | 10.6% | 100.0% |
| GLM Fixed eff | 99.3% | -2.4% | 13.6% | 99.5% | 98.9% | -2.9% | 14.2% | 99.2% | 99.4% | -2.5% | 14.2% | 99.3% |
| GLM Rand eff | 100.0% | -9.5% | 16.1% | 40.3% | 100.0% | -9.5% | 16.2% | 41.1% | 100.0% | -9.3% | 16.2% | 40.8% |
|  |  |  |  |  |  |  |  |  |  |  |  |  |
| Number of LYs |  |  |  |  |  |  |  |  |  |  |  |  |
| Direct comparison | 98,9% | -5,7% | 21,7% | 81,5% | 97,7% | -6,6% | 21,9% | 80,6% | 97.8% | -6.0% | 21,9% | 81,0% |
| Song | 98,4% | -3,0% | 17,9% | 95,3% | 97,5% | -4,7% | 17,8% | 93,5% | 98.0% | -7.8% | 18.8% | 93.1% |
| Puhan | 97,2% | -2,1% | 16,9% | 99,3% | 96,7% | -2,7% | 16,9% | 98,9% | 96.6% | -6.2% | 17.6% | 98.3% |
| GLM Fixed eff | 99.6% | -3.5% | 22.7% | 73.4% | 98.9% | -4.0% | 23.5% | 71.2% | 99.6% | -3.5% | 23.3% | 71.4% |
| GLM Rand eff | 100.0% | -13.6% | 25.9% | 5.8% | 100.0% | -13.4% | 25.9% | 5.6% | 100.0% | -13.4% | 26.0% | 6.2% |
|  |  |  |  |  |  |  |  |  |  |  |  |  |
| Number of Events |  |  |  |  |  |  |  |  |  |  |  |  |
| Direct comparison | 98,4% | -0,4% | 6,0% | 100,0% | 98,3% | -0,3% | 5,9% | 100,0% | 99.6% | -0.1% | 7,0% | 100,0% |
| Song | 98,4% | -0,2% | 5,1% | 100,0% | 98,7% | -0,1% | 4,9% | 100,0% | 99.1% | -1.0% | 5.1% | 100.0% |
| Puhan | 97,0% | 0,2% | 4,9% | 100,0% | 97,3% | 0,3% | 4,8% | 100,0% | 99.0% | -0.5% | 4.9% | 100.0% |
| GLM Fixed eff | 98.7% | -0.3% | 6.2% | 100.0% | 98.9% | -0.4% | 6.1% | 100.0% | 99.6% | -0.2% | 6.1% | 100.0% |
| GLM Rand eff | 100.0% | -2.7% | 6.9% | 90.6% | 100.0% | -2.8% | 6.6% | 92.4% | 100.0% | -2.8% | 6.7% | 90.9% |
|  |  |  |  |  |  |  |  |  |  |  |  |  |
| Total costs |  |  |  |  |  |  |  |  |  |  |  |  |
| Direct comparison | 99,0% | 0,4% | 8,0% | 100,0% | 99,1% | 0,1% | 7,8% | 100,0% | 99.3% | 0.1% | 8,2% | 100,0% |
| Song | 98,6% | 0,8% | 6,6% | 100,0% | 98,7% | 0,5% | 6,3% | 100,0% | 99.2% | 0.3% | 6.6% | 100.0% |
| Puhan | 97,3% | 0,8% | 6,3% | 100,0% | 97,7% | 0,7% | 6,1% | 100,0% | 98.6% | 0.4% | 6.2% | 100.0% |
| GLM Fixed eff | 99.7% | 0.3% | 8.4% | 100.0% | 99.3% | 0.4% | 8.3% | 100.0% | 99.6% | 0.2% | 8.2% | 100.0% |
| GLM Rand eff | 100.0% | 0.3% | 8.9% | 95.9% | 100.0% | 0.3% | 8.5% | 96.1% | 100.0% | 0.2% | 8.4% | 94.8% |

Table A9: Health-economic outcomes for two of the eight scenarios. Means of coverage, bias and mean absolute deviance (MAD) of the difference between two interventions, over 1,000 repetitions.

| Scenario | Scenario 4 | | | | Scenario 5 | | | |
| --- | --- | --- | --- | --- | --- | --- | --- | --- |
|  | Eight randomly drawn trials; one trial drawn from a less healthy population | | | | Seven randomly drawn trials; one trial drawn from a less healthy population; one from a relatively younger population | | | |
|  | Coverage | Bias | MAD | Stat Power | Coverage | Bias | MAD | Stat Power |
| Number of QALYs |  |  |  |  |  |  |  |  |
| Direct comparison | 97,1% | -8,4% | 14,4% | 98,7% | 98,6% | -3,5% | 13,1% | 99,8% |
| Song | 98,3% | -4,1% | 11,2% | 100,0% | 99,2% | -2,2% | 10,5% | 100,0% |
| Puhan | 96,8% | -4,0% | 10,5% | 100,0% | 97,7% | -1,1% | 9,9% | 100,0% |
| GLM Fixed eff | 99.2% | -7.0% | 15.2% | 97.9% | 99.5% | -2.0% | 13.8% | 99.5% |
| GLM Rand eff | 100.0% | -13.6% | 18.5% | 34.5% | 100.0% | -9.0% | 15.8% | 43.6% |
|  |  |  |  |  |  |  |  |  |
| Number of LYs |  |  |  |  |  |  |  |  |
| Direct comparison | 97,1% | -11,8% | 23,5% | 76,3% | 98,1% | -5,2% | 21,9% | 82,7% |
| Song | 98,9% | -5,5% | 18,4% | 93,5% | 98,5% | -3,5% | 17,8% | 95,1% |
| Puhan | 97,1% | -5,4% | 17,4% | 98,6% | 97,1% | -1,6% | 16,7% | 99,2% |
| GLM Fixed eff | 99.3% | -9.3% | 25.1% | 56.8% | 99.2% | -2.6% | 22.9% | 74.0% |
| GLM Rand eff | 100.0% | -18.9% | 29.2% | 4.1% | 100.0% | -12.4% | 25.2% | 7.7% |
|  |  |  |  |  |  |  |  |  |
| Number of Events |  |  |  |  |  |  |  |  |
| Direct comparison | 98,1% | -2,6% | 6,6% | 100,0% | 98,9% | -0,5% | 6,3% | 100,0% |
| Song | 98,1% | -0,8% | 5,3% | 100,0% | 99,2% | -0,1% | 5,1% | 100,0% |
| Puhan | 97,8% | -0,7% | 5,1% | 100,0% | 99,0% | 0,2% | 5,1% | 100,0% |
| GLM Fixed eff | 99.6% | -2.2% | 6.8% | 100.0% | 99.2% | -0.5% | 6.5% | 100.0% |
| GLM Rand eff | 100.0% | -4.7% | 7.9% | 93.1% | 100.0% | -3.0% | 7.0% | 93.1% |
|  |  |  |  |  |  |  |  |  |
| Total costs |  |  |  |  |  |  |  |  |
| Direct comparison | 98,6% | -0,5% | 8,3% | 100,0% | 98,2% | 0,7% | 8,2% | 100,0% |
| Song | 99,0% | -0,5% | 6,6% | 100,0% | 98,6% | 0,7% | 6,6% | 100,0% |
| Puhan | 97,9% | -0,5% | 6,4% | 100,0% | 97,7% | 1,0% | 6,5% | 100,0% |
| GLM Fixed eff | 99.6% | -0.8% | 9.0% | 100.0% | 99.2% | 0.9% | 8.6% | 100.0% |
| GLM Rand eff | 100.0% | -0.7% | 9.4% | 94.3% | 100.0% | 1.0% | 8.9% | 96.4% |

Table A10: Health-economic outcomes for three of the eight scenarios. Means of coverage, bias and mean absolute deviance (MAD) of the difference between two interventions, over 1,000 repetitions.

| Scenario | Scenario 6 | | | | Scenario 7 | | | | Scenario 8 | | | |
| --- | --- | --- | --- | --- | --- | --- | --- | --- | --- | --- | --- | --- |
|  | Six randomly drawn trials; one trial drawn from a less healthy population; one from a relatively younger population; one from a relatively older population | | | | Six randomly drawn trials; three trials drawn from a less healthy population | | | | Six randomly drawn trials; three trials drawn from a less healthy population (extreme scenario) | | | |
|  | Coverage | Bias | MAD | Stat Power | Coverage | Bias | MAD | Stat Power | Coverage | Bias | MAD | Stat Power |
| Number of QALYs |  |  |  |  |  |  |  |  |  |  |  |  |
| Direct comparison | 97,4% | -6,0% | 13,9% | 99,6% | 97,2% | -8,0% | 14,3% | 99,3% | 98.9% | -11.9% | 15.5% | 99.3% |
| Song | 98,1% | -3,9% | 11,0% | 100,0% | 97,9% | -7,5% | 12,0% | 99,9% | 99.6% | -11.2% | 13.7% | 99.5% |
| Puhan | 96,5% | -2,8% | 10,2% | 100,0% | 96,3% | -6,5% | 11,2% | 100,0% | 90.7% | -9.8% | 12.3% | 100.0% |
| GLM Fixed eff | 99.1% | -4.7% | 14.6% | 99.1% | 99.4% | -6.6% | 15.0% | 97.4% | 100.0% | -10.5% | 18.3% | 73.0% |
| GLM Rand eff | 100.0% | -11.2% | 17.3% | 37.4% | 100.0% | -13.5% | 18.7% | 32.2% | 100.0% | -16.3% | 21.4% | 10.0% |
|  |  |  |  |  |  |  |  |  |  |  |  |  |
| Number of LYs |  |  |  |  |  |  |  |  |  |  |  |  |
| Direct comparison | 97,0% | -8,8% | 23,0% | 79,7% | 97,4% | -10,1% | 22,8% | 79,3% | 99.1% | -11.2% | 22.5% | 70.0% |
| Song | 97,9% | -5,4% | 18,5% | 92,9% | 98,5% | -9,7% | 19,2% | 91,9% | 99.2% | -11.9% | 19.9% | 83.7% |
| Puhan | 96,7% | -3,8% | 17,2% | 98,7% | 96,9% | -8,1% | 18,0% | 98,0% | 99.0% | -9.3% | 18.0% | 94.1% |
| GLM Fixed eff | 98.9% | -6.4% | 24.3% | 63.9% | 99.2% | -7.7% | 24.1% | 60.3% | 100.0% | -9.2% | 27.6% | 13.0% |
| GLM Rand eff | 100.0% | -16.0% | 27.5% | 4.7% | 100.0% | -17.8% | 28.5% | 3.6% | 100.0% | -17.5% | 31.0% | 0.5% |
|  |  |  |  |  |  |  |  |  |  |  |  |  |
| Number of Events |  |  |  |  |  |  |  |  |  |  |  |  |
| Direct comparison | 98,4% | -0,8% | 6,1% | 100,0% | 98,8% | -3,1% | 6,3% | 100,0% | 98.3% | -10.0% | 10.6% | 100.0% |
| Song | 98,1% | -0,7% | 5,4% | 100,0% | 98,4% | -1,9% | 5,4% | 100,0% | 99.4% | -5.8% | 7.3% | 100.0% |
| Puhan | 97,7% | -0,2% | 5,1% | 100,0% | 97,6% | -1,5% | 5,1% | 100,0% | 90.1% | -6.0% | 7.2% | 100.0% |
| GLM Fixed eff | 99.0% | -0.8% | 6.3% | 100.0% | 99.3% | -2.8% | 6.8% | 100.0% | 99.8% | -8.4% | 10.4% | 100.0% |
| GLM Rand eff | 100.0% | -3.3% | 7.1% | 89.9% | 100.0% | -5.3% | 7.9% | 88.3% | 100.0% | -11.7% | 13.1% | 73.4% |
|  |  |  |  |  |  |  |  |  |  |  |  |  |
| Total costs |  |  |  |  |  |  |  |  |  |  |  |  |
| Direct comparison | 98,3% | -1,0% | 8,1% | 100,0% | 99,1% | 0,2% | 7,8% | 100,0% | 99.7% | 3.1% | 8.3% | 100.0% |
| Song | 98,9% | -0,3% | 7,0% | 100,0% | 99,3% | -0,5% | 6,6% | 100,0% | 100.0% | -0.4% | 6.9% | 100.0% |
| Puhan | 97,1% | -0,3% | 6,6% | 100,0% | 98,2% | -0,4% | 6,4% | 100,0% | 91.6% | 0.5% | 6.4% | 100.0% |
| GLM Fixed eff | 99.5% | -1.0% | 8.7% | 100.0% | 99.9% | 0.0% | 8.7% | 100.0% | 100.0% | 1.7% | 10.1% | 99.8% |
| GLM Rand eff | 100.0% | -1.1% | 9.0% | 92.6% | 100.0% | -0.2% | 8.9% | 93.5% | 100.0% | 2.5% | 10.7% | 83.2% |
