# Supplementary material for: Mix and match. A simulation study on the impact of mixed-treatment comparison methods on health-economic outcomes
Source: PLoS One. 2017 Feb 2;12(2):e0171292. doi: 10.1371/journal.pone.0171292 (PMC5289594; doi:10.1371/journal.pone.0171292)
Supplement: S1 Text — (DOCX) [file pone.0171292.s005.docx]

**S1 Text Converting Odds Ratio to Relative Risk**

For Puhan’s method, GLMFE and GLMRE, the odds ratio (OR) is calculated as a relative measure between treatment arms. Most HE models are, however, based on the relative risk (RR) or the risk difference (RD). In this appendix we show how the RR, RD and their respective variances are calculated, using the baseline estimates for the control arm and the estimated OR from the MTC.

The baseline estimate for the control arm ($\hat{\delta}_{c}$) can be written as $\hat{\delta}_{c}={rc}/{nc}$, where rc is the number of “successes” in the control arm, and nc is the total number of people in the control arm. The estimated variance of $\hat{\delta}_{c}$ can be written as ${Var}_{\hat{\delta}_{c}}=\frac{\hat{\delta}_{c}\left( 1-\hat{\delta}_{c} \right)}{nc}$.

With some basic manipulation, one can estimate both rc and nc:

$\hat{nc}=\frac{\hat{\delta}_{c}\left( 1-\hat{\delta}_{c} \right)}{{Var}_{\hat{\delta}_{c}}}$ (S1 Eq)

$\hat{rc}=\hat{\delta}_{c}*\hat{nc}$ (S2 Eq)

From the MTC, we have an estimate of the odds ratio $\hat{\delta}_{OR}$, and the variance of $Ln(\hat{\delta}_{OR})$, which can also be written in terms of the number of successes (rc for the control arm, and rt for the treatment arm) and the total number of people in both arms (nc and nt, respectively).

$\hat{\delta}_{OR}=\frac{rt\left( nc-rc \right)}{rc\left( nt-rt \right)}$ (S3 Eq)

${Var}_{Ln(\hat{\delta}_{OR})}=\frac{1}{rc}+\frac{1}{\left( nc-rc \right)}+\frac{1}{rt}+\frac{1}{\left( nt-rt \right)}$ (S4 Eq)

Using $\hat{nc}$ and $\hat{nt}$, we can write, from (S3 Eq):

$\hat{\delta}_{OR}=\frac{rt\left( \hat{nc}-\hat{rc} \right)}{\hat{rc}\left( nt-rt \right)}=\frac{\left( \hat{nc}-\hat{rc} \right)}{\hat{rc}}\frac{rt}{\left( nt-rt \right)}$ (S5 Eq)

$\frac{rt}{\left( nt-rt \right)}=\delta_{OR}\frac{\hat{rc}}{\left( \hat{nc}-\hat{rc} \right)}$ (S6 Eq)

From (S4 Eq):

$\frac{1}{rt}+\frac{1}{\left( nt-rt \right)}={Var}_{Ln\left( \hat{\delta}_{OR} \right)}-\frac{1}{\hat{rc}}-\frac{1}{\left( \hat{nc}-\hat{rc} \right)}$ (S7 Eq)

$\frac{rt}{rt}+\frac{rt}{\left( nt-rt \right)}=rt\left[ {Var}_{Ln\left( \hat{\delta}_{OR} \right)}-\frac{1}{\hat{rc}}-\frac{1}{\left( \hat{nc}-\hat{rc} \right)} \right]$ (S8 Eq)

Combining (S6) and (S8):

$1+\hat{\delta}_{OR}\frac{\hat{rc}}{\left( \hat{nc}-\hat{rc} \right)}=rt\left[ {Var}_{Ln\left( \hat{\delta}_{OR} \right)}-\frac{1}{\hat{rc}}-\frac{1}{\left( \hat{nc}-\hat{rc} \right)} \right]$ (S9 Eq)

From this we can estimate $\hat{rt}$:

$\hat{rt}=\frac{\left[ 1+\hat{\delta}_{OR}\frac{\hat{rc}}{\left( \hat{nc}-\hat{rc} \right)} \right]}{\left[ {Var}_{Ln\left( \hat{\delta}_{OR} \right)}-\frac{1}{\hat{rc}}-\frac{1}{\left( \hat{nc}-\hat{rc} \right)} \right]}$ (S10 Eq)

Substituting the estimate $\hat{rt}$ in (S6 Eq), we can estimate $\hat{nt}$:

$\frac{\left( nt-\hat{rt} \right)}{\left( \hat{rt} \right)}=\left[ \hat{\delta}_{OR}\frac{\hat{rc}}{\left( \hat{nc}-\hat{rc} \right)} \right]$ (S11)

$nt-\hat{rt}=\hat{rt}\left[ \hat{\delta}_{OR}\frac{\hat{rc}}{\left( \hat{nc}-\hat{rc} \right)} \right]$ (S12)

$\hat{nt}=\hat{rt}\left[ \hat{\delta}_{OR}\frac{\hat{rc}}{\left( \hat{nc}-\hat{rc} \right)} \right]+\hat{rt}$ (S13)

Both the risk ratio and the risk difference, and their respective variances are easily then estimated using $\hat{nc}$, $\hat{rc}$, $\hat{nt}$, $\hat{rt}$ and these formulas:

$\hat{\delta}_{RR}=\frac{\hat{rt}/\hat{nt}}{\hat{rc}/\hat{nt}}$, ${Var}_{{Ln(\hat{\delta}}_{RR})}=\frac{1}{\hat{rc}}+\frac{1}{\hat{nc}}+\frac{1}{\hat{rt}}+\frac{1}{\hat{nt}}$ (S14 Eq, S15 Eq)

$\hat{\delta}_{RD}=\frac{\hat{rt}}{\hat{nt}}-\frac{\hat{rc}}{\hat{nc}}$, ${Var}_{\hat{\delta}_{RD}}=\left( \frac{\hat{rt}}{\hat{nt}} \right)^{2}+\left( \frac{\hat{rc}}{\hat{nc}} \right)^{2}$ (S16 Eq, S17 Eq)
